# Supplementary material for: P-TRAP: a Panicle Trait Phenotyping tool
Source: BMC Plant Biol. 2013 Aug 29;13:122. doi: 10.1186/1471-2229-13-122 (PMC3848748; doi:10.1186/1471-2229-13-122)
Supplement: Additional file 7 — P-TRAP source code. The Netbeans project that contains the source code of the application. [file 1471-2229-13-122-S7.zip › javadoc.zip!/javadoc/org-bioalg-imageprocessor/org/bioalg/imageprocessor/EDistanceMap.html]

EDistanceMap (Image Processor)


JavaScript is disabled on your browser.


- Package
- Class
- Tree
- Deprecated
- Index
- Help

*org.bioalg.imageprocessor 1.0*

- Prev Class
- Next Class

- Frames
- No Frames

- All Classes

- Summary:
- Nested |
- Field |
- Constr |
- Method

- Detail:
- Field |
- Constr |
- Method


org.bioalg.imageprocessor

## Class EDistanceMap

- java.lang.Object
- - org.bioalg.imageprocessor.EDistanceMap

- ---

    

  ```
  public abstract class EDistanceMap
  extends java.lang.Object
  ```

  The distance mapper of the image.
  PAPER A. Meijster, J.B.T.M. Roerdink, and W.H. Hesselink "A General
  Algorithm for Computing Distance Transforms in Linear Time", proc. of 5th
  Int. Conf. Mathematical Morphology and its Applications to Image and
  Signal Processing, 2000
  ported from this paper's c++ code by Faroq.

- - ### Constructor Summary

    Constructors

    Constructor and Description

    `EDistanceMap()`
  - ### Method Summary

    Methods

    | Modifier and Type | Method and Description |
    | --- | --- |
    | `static AImage` | `EDT_1D(AImage source)` Perform the first step of 1D Distance mapping |
    | `static AImage` | `meijster(AImage source)` The meijster EDM algorithm |
    | `static double` | `meijsterFunction(int x, int i, double g)` |

    - ### Methods inherited from class java.lang.Object

      `clone, equals, finalize, getClass, hashCode, notify, notifyAll, toString, wait, wait, wait`

- - ### Constructor Detail


    - #### EDistanceMap

      ```
      public EDistanceMap()
      ```
  - ### Method Detail


    - #### meijsterFunction

      ```
      public static double meijsterFunction(int x,
                            int i,
                            double g)
      ```


    - #### meijster

      ```
      public static AImage meijster(AImage source)
      ```

      The meijster EDM algorithm

      Parameters:
      :   `source` - a binary `AImage`

      Returns:
      :   the distance map image.


    - #### EDT\_1D

      ```
      public static AImage EDT_1D(AImage source)
      ```

      Perform the first step of 1D Distance mapping

      Parameters:
      :   `source` - a binary `AImage`

      Returns:
      :   the 1D map of the image.


- Package
- Class
- Tree
- Deprecated
- Index
- Help

*org.bioalg.imageprocessor 1.0*

- Prev Class
- Next Class

- Frames
- No Frames

- All Classes

- Summary:
- Nested |
- Field |
- Constr |
- Method

- Detail:
- Field |
- Constr |
- Method
